# Conscious rat PET imaging with soft immobilization for quantitation of brain functions: comprehensive assessment of anesthesia effects on cerebral blood flow and metabolism

**DOI:** 10.1186/s13550-021-00787-6

**Published:** 2021-05-08

**Authors:** Chie Suzuki, Mutsumi Kosugi, Yasuhiro Magata

**Affiliations:** Preeminent Medical Photonics Education and Research Center, Hamamatsu University School of Medicine, 1-20-1 Handayama, Higashi-ku, Hamamatsu, Shizuoka 431-3192 Japan

**Keywords:** Cerebral blood flow and metabolism, Consciousness, Positron emission tomography (PET), Rat, Three types of mixed anesthetic agents

## Abstract

**Background:**

Animal brain functions evaluated by in vivo imaging under anesthesia can be affected by anesthetic agents, resulting in incorrect assessment of physiological brain function. We therefore performed dynamic positron emission tomography (PET) imaging of conscious rats using recently reported soft immobilization to validate the efficacy of the immobilization for brain function assessments. We also determined the effects of six anesthetic agents—a mixed anesthetic agent (MMB), ketamine + xylazine (KX), chloral hydrate (Chloral), pentobarbital (PTB), propofol (PF), and isoflurane (IFL)—on brain function by comparison with conscious rats.

**Results:**

The immobilization enabled 45-min dynamic [^18^F]FDG-PET acquisition with arterial blood sampling using conscious rats without the use of special techniques or invasive surgery. The spatial resolution and quantitativity of [^18^F]FDG-PET were not significantly lower for conscious rats than for anesthetized rats. While MMB, Chloral, PTB, and PF showed ubiquitous reduction in the cerebral metabolic rates of glucose (CMR_glu_) in brain regions, KX and IFL showed higher reductions in cerebellum and interbrain, and cerebellum, respectively. Cerebral blood flow (CBF) was reduced by MMB, KX, PTB, and PF; increased by IFL; and unaltered by Chloral. The magnitude of decrease in CMR_glu_ and CBF for MMB were not larger than for other five anesthetic agents, although blood glucose levels and body temperature can be easily affected by MMB.

**Conclusion:**

The six anesthetic agents induced various effects on CMR_glu_ and CBF. The immobilization technique presented here is a promising tool for noninvasive brain functional imaging using conscious rats to avoid the effects of anesthetic agents.

**Supplementary Information:**

The online version contains supplementary material available at 10.1186/s13550-021-00787-6.

## Background

In vivo imaging techniques, such as positron emission tomography (PET), single photon emission computed tomography (SPECT), and magnetic resonance imaging (MRI), are powerful tools for estimating brain function in living animals. Anesthetic agents are generally used for animal imaging to prevent motion artifacts caused by animal movement during the scan. Anesthetic agents have various significant effects on brain function, including cerebral metabolism [1] and neural activities [2]. Therefore, animal brain functions that are evaluated by in vivo imaging under anesthesia could be affected by anesthetic agents, resulting in incorrect assessment of brain functions under physiological conditions.

To evaluate animal brain functions without the effect of anesthesia, several imaging techniques have been reported. In one approach, PET/SPECT imaging for estimating conscious animal brain functions, conscious animals are administrated radiotracers and kept conscious during tracer distribution [3, 4]. At the appropriate time [~ 40–60 min for 2-deoxy-2-[^18^F]fluoroglucose ([^18^F]FDG)] post-injection, animals are anesthetized and a static PET/SPECT scan is performed under anesthesia. This approach is applicable only in limited probes that show fast distribution according to the brain functions and stabilize their concentration in the regions of interest (ROIs) that are not affected by anesthetic agents during the scanning. Another approach for imaging conscious animals is using a head holder to prevent head movement [5–7]. Because head holders are often directly attached to animal skulls by invasive surgical procedures, the effects on the animals’ physiological condition from attachment of the head holder should be considered. Additionally, the gamma-ray scattering and attenuation caused by the head holders should be considered. Reconstruction techniques for free moving animals with motion correction using motion tracking systems have also been reported [8–10]. However, these techniques have not been able to be widely used yet, since they require additional hardware and/or complicated software. Recently, soft immobilization for conscious mouse MRI scanning has been reported [11]. Because this immobilization, which uses a simple soft restrainer, suppresses the head movements of conscious mice for at least 30 min without surgery or a metallic apparatus [11], it could be suitable for dynamic PET scanning.

In this study, we assessed the cerebral metabolic rate of glucose (CMR_glu_) and cerebral blood flow (CBF) in conscious rats using the soft immobilization technique, and validated its efficacy for brain function imaging of conscious rats. In addition, we also determined the effects of six anesthetic agents on CMR_glu_ and CBF by comparison with conscious rats.

## Methods

### General

Fluorine-18 was produced by ^18^O(p, n)^18^F nuclear reaction using a 12 MeV cyclotron (HM-12, Sumitomo Heavy Industry, Tokyo, Japan). [^18^F]FDG was radiosynthesized using a [^18^F]FDG synthesizer F200 (Sumitomo Heavy Industries). The radiochemical purity of [^18^F]FDG, determined by thin-layer chromatography (Tec-Control chromatography system, Biodex, NY, USA), was higher than 95%. [^125^I]IMP was supplied by Nihon Medi-Physics Co., Ltd. (Tokyo, Japan). Male Sprague–Dawley rats (Slc:SD) were supplied by Japan SLC Co. (Hamamatsu, Japan) and housed under a 12-h light/12-h dark cycle with free access to food and water. The animal experiments were performed in accordance with institutional and national guidelines regarding animal care, and were approved by the Animal Care and Use Committee of the Hamamatsu University School of Medicine.

### Animal preparation

Rats aged 8–9 weeks old were used for the experiments, and their weights ranged from 233.8 to 339.4 g (277.2 ± 24.4 g). Rats (*n* = 6 or 7) were fasted for 15 h from the evening before and then anesthetized with the anesthetic agents described below. A polyethylene catheter (i.d. 0.5 mm, o.d. 0.8 mm) was inserted into the femoral artery for blood sampling and filled with heparin saline solution (10 IU/mL), and an indwelling needle (Surflo I.V. Catheter 24 G, SR-OT2419C, Terumo Co., Tokyo, Japan) was inserted into the tail vein for the administration of radiotracers and continuous infusion of propofol. These cannulas were in place until euthanasia. Body temperature was maintained at approximately 37 °C by a heating pad during the preparation. Rats were restrained on the bed and a head CT scan was performed using a small animal PET/SPECT/CT system (FLEX; Gamma Medica Ideas, Northridge, CA, USA). Blood gas profiles and glucose levels in arterial blood were measured using an i-STAT analyzer with an i-STAT CG8 + cartridge test (Abbot Point of Care, Illinois, USA) 5 min before [^18^F]FDG injection.

### Handmade restrainer

**Fig. 1:**
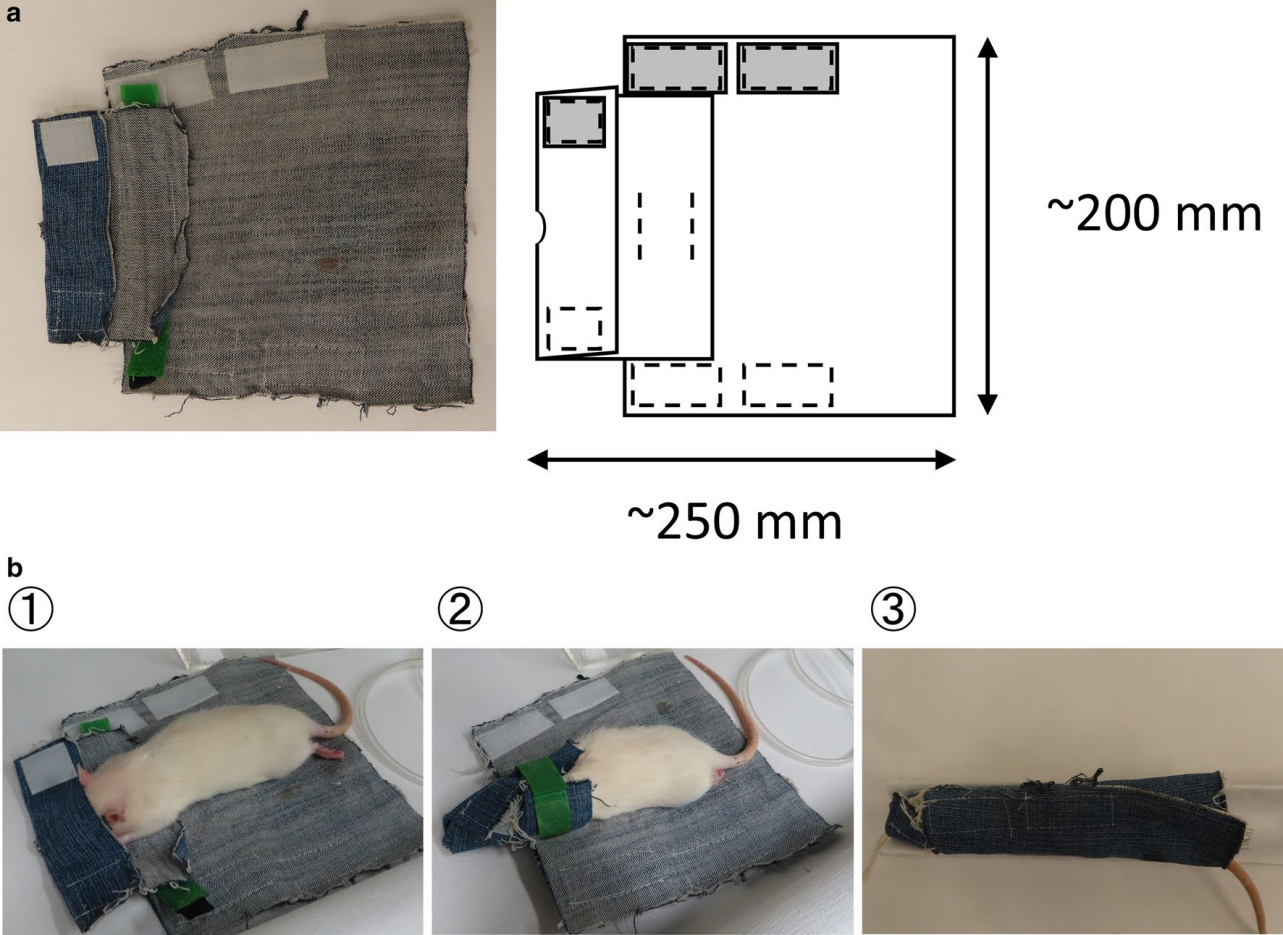
Soft immobilization of conscious rats. **a** Photograph (left) and drawing (right) of the handmade restrainer. Dashed lines in the drawing show the stitch. **b** The rat was temporarily anesthetized and restrained so that PET imaging could be performed after recovering consciousness. (1) The rat head was covered with the hood. (2) The head and neck were held by hook-and-loop fasteners. (3) The whole body was wrapped in the restrainer

Two square pieces of denim fabric (200 mm × 200 mm and 160 mm × 160 mm) were sewn together and folded to make a hood. A small cut was made in the hood to create an air vent. Several hook-and-loop fasteners were attached to hold the head, neck, and abdomen of the rats (Fig. 1a).

### Anesthetic conditions

Conscious (CONS): To allow the rats to acclimate to the restrainer, the restrainer was applied before being used for the experiments, as described below. Rats were anesthetized with an inhalation of 3% isoflurane (ISOFLURANE Inhalation Solution, Mylan, PA, USA) in 50% oxygen (1 L/min), and wrapped in the handmade restrainer (Fig. 1b) within 5 min. The rats were then restrained on a bed for 75 min without isoflurane inhalation. This acclimation was repeated once daily every weekday at least nine times.

On the experiment day, rats were anesthetized with an inhalation of 3% isoflurane in 50% oxygen (1 L/min) during arterial and venous cannulation (< 30 min), and lidocaine hydrochloride (Xylocaine Jelly 2%, Aspen Japan KK, Tokyo, Japan) was applied to the incision. After cannulation, rats were wrapped in the restrainer, and restrained for 30 min to induce emergence from isoflurane.

Medetomidine, midazolam, and butorphanol (MMB) [12]: Rats were intraperitoneally injected with a mixture of 0.375 mg/kg medetomidine hydrochloride (Domitol, Nippon Zenyaku Kogyo Co., Ltd., Fukushima, Japan), 2 mg/kg midazolam (Dormicum, Astellas Pharma Inc., Tokyo, Japan), and 2.5 mg/kg butorphanol (Vetorphale, Meiji Seika Pharma Co., Ltd., Tokyo, Japan).

Ketamine + xylazine (KX): Rats were intraperitoneally injected with a mixture of 80 mg/kg ketamine hydrochloride (Ketalar, Sankyo Lifetech Co., Ltd., Tokyo, Japan) and 8 mg/kg xylazine hydrochloride (Celactar, Bayer, Ltd., Tokyo, Japan).

Chloral hydrate (Chloral): Rats were intraperitoneally injected with 3.5% chloral hydrate (FUJIFILM Wako Pure Chemical Co., Osaka, Japan) solution in saline (400 mg/kg).

Pentobarbital (PTB): Rats were intraperitoneally injected with 50 mg/kg sodium pentobarbital (Somnopentyl, Kyoritsu Seiyaku Co., Tokyo, Japan).

Propofol (PF): Anesthesia was initially induced by intravenous injection of 10 mg/kg propofol (1%, Maruishi Pharmaceutical. Co., Ltd. Osaka, Japan), and then maintained by intravenous infusion of 20 mg/kg/h propofol via the intravenous cannula, except during radiotracer injection (~ 30 s each).

Isoflurane (IFL): Anesthesia was initially induced by inhalation of 4% isoflurane in 50% oxygen (1 L/min), and then maintained by inhalation of 1.5% isoflurane in 50% oxygen (1 L/min).

### Experimental protocol

[^18^F]FDG (~ 37 MBq) dissolved in 0.5 mL of saline was injected intravenously into the rats. Immediately after the [^18^F]FDG injection, dynamic PET scans were performed for 45 min (6 × 30 s and 42 × 60 s) using a PET scanner HITS-655 K (Hamamatsu Photonics KK, Hamamatsu, Japan) [13]. Arterial blood (approximately 100 μL per sample) was collected at 5, 15, 25, 35, 45, 55, 90, 120, 300, 450, 600, 1500, 2100, and 2700 s post-injection of [^18^F]FDG, and radioactivity concentration in the plasma was measured using an auto-well γ counter (1480 WIZARD^2^ 3, PerkinElmer, Waltham, MA, USA). Heparinized saline was injected via the arterial cannula to avoid hypovolemia and cannula embolization at the interval of arterial blood sampling. PET images were reconstructed by list-mode dynamic row action maximum likelihood algorithm (LM-DRAMA) with four iterations without attenuation and scatter corrections [13]. The ROIs were manually drawn over the whole brain and over several brain regions (cerebellum, hippocampus, striatum, cortex, thalamus, and hypothalamus) by referring to normal rat brain MRI images, and radioactivity in the ROIs was quantified as the mean concentration. [^18^F]FDG brain accumulation was presented as standardized uptake value (SUV): SUV = tissue radioactivity concentration (Bq/mL)/injected dose (Bq) × body weight (g). Index values, attained by multiplying the brain/plasma ratio by blood glucose levels (BPG), were calculated as follows: BPG (mmol/L) = brain radioactivity concentration (Bq/mL) at 44–45 min post-injection of [^18^F]FDG/plasma radioactivity concentration (Bq/mL) at 45 min post-injection of [^18^F]FDG × blood glucose levels (mmol/L). [^18^F]FDG kinetics were analyzed by the 2-tissue-3-compartment model using arterial plasma ^18^F radioactivity concentration as an input function to obtain uptake rate constants (*K*_1_; mL/min/g), efflux rate constants (*k*_2_; /min), phosphorylation reaction constants (*k*_3_; /min), and dephosphorylation reaction constants (*k*_4_; /min) using Microsoft Excel Solver (Microsoft Corp., Redmond, WA) [14]. We used a single lumped constant (0.625 [15]) for the different anesthetized groups for CMR_glu_ calculation as the detailed effects of anesthetic agents on the lumped constant have not been elucidated.

After PET scanning, the rats were intravenously injected with [^125^I]IMP (~ 185 kBq), and arterial blood (approximately 50 µL per sample) was collected at 2, 6, 10, 14, 18, 22, 26, 30, 34, 38, 42, 46, 50, 54, 58, 100, 120, 150, 180, 210, 240, and 300 s post-injection of [^125^I]IMP. At 5-min post-injection of [^125^I]IMP, the rats were immediately sacrificed by decapitation, and the brain was removed and weighed. ^125^I radioactivity concentrations in the arterial blood samples and ^18^F and ^125^I radioactivity concentrations in the brain tissues were measured with an auto-well γ counter. The CBF value was calculated as previously described [16] using 0.92 [17] as the first-pass extraction fraction of IMP and 1.0 (0–26.5 s), 0.8 (26.5–51.5 s), and 0.75 (51.5–300 s) as the octanol extraction ratios, indicating the ratio of intact [^125^I]IMP in each blood sample [18].

### Corticosterone assay

To monitor the stress caused by restraining and/or blood sampling during PET acquisition, plasma corticosterone levels were measured. Arterial blood (200 µL) was collected for corticosterone assay from conscious rats and MMB- and IFL-anesthetized rats (before [^18^F]FDG injection, *n* = 4). Five-minutes later, these rats were intravenously injected with 0.5 mL of saline instead of [^18^F]FDG, and arterial blood sampling was performed as described above. After arterial blood sampling at 2700 s post-injection, additional arterial blood (200 µL) was collected for the corticosterone assay (after [^18^F]FDG-PET). Next, 0.5 mL of saline instead of [^125^I]IMP was intravenously injected and arterial blood sampling was performed as described above. After arterial blood sampling at 300 s post-injection, additional arterial blood (200 µL) was collected for the corticosterone assay (after [^125^I]IMP assay). Arterial blood samples for the corticosterone assay were centrifuged at 1500×*g* at 4 °C for 5 min to prepare plasma, which was stored at − 80 °C until the assay. The plasma corticosterone levels were measured using an ELISA kit (Enzo Life Science, ADI-900-097, PA).

### Statistical analysis

Data are expressed as mean ± SD. The statistical significance of the differences between the conscious and anesthetized rats was determined using non-repeated ANOVA with Dunnett’s multiple comparison test. The statistical significance of the differences in plasma corticosterone levels between before [^18^F]FDG injection, after [^18^F]FDG-PET, and after [^125^I]IMP assay was determined using repeated ANOVA with Tukey’s multiple comparison test. *P* values of less than 0.05 were considered statistically significant. The association between SUV determined by PET and that determined by γ counter was calculated by Pearson’s product moment correlation coefficient. Differences between regression coefficients of the regression lines were determined by testing the *t*-value. The association between CMR_glu_ and brain SUV or BPG were also calculated by Pearson’s product moment correlation coefficient.

## Results

### Animal condition during experiments and quality of PET data of conscious rats

**Fig. 2:**
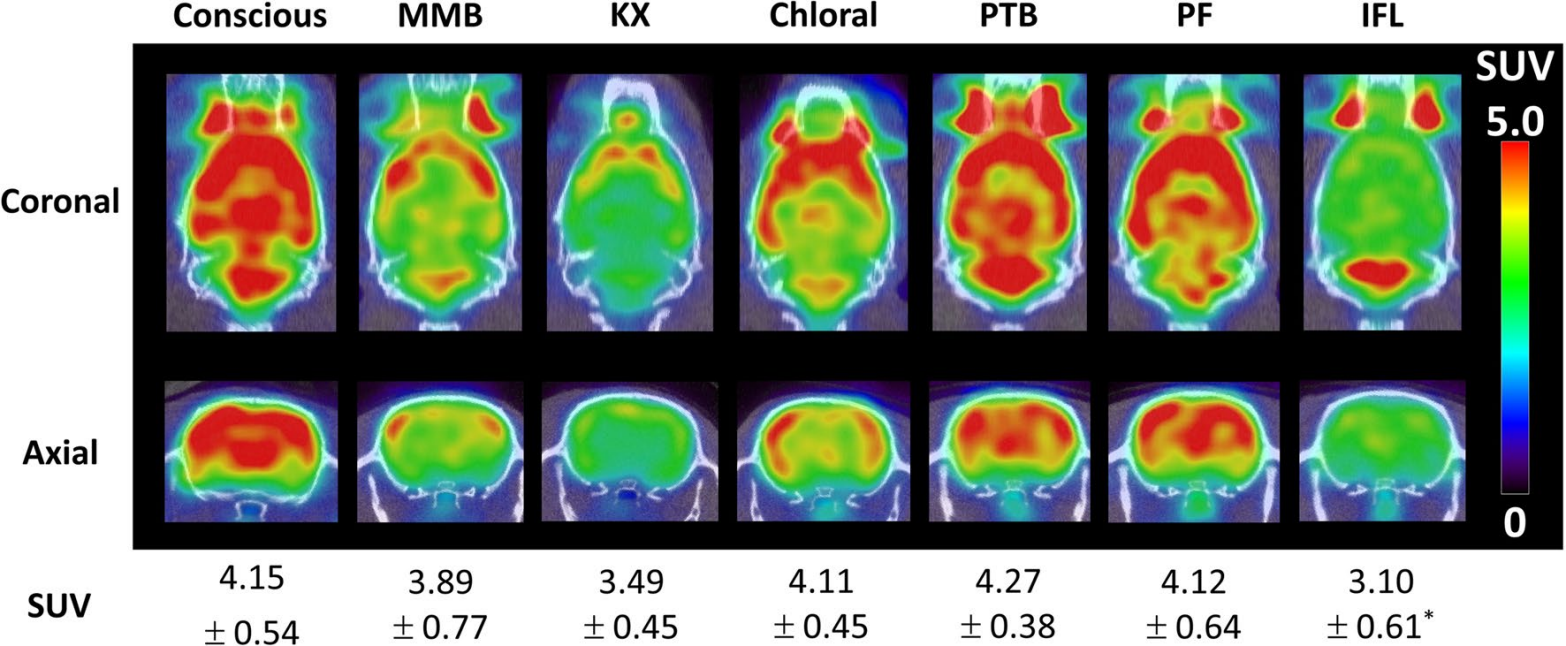
Brain [^18^F]FDG-PET uptake in conscious and anesthetized rats. Typical [^18^F]FDG-PET images and brain standardized uptake value (SUV, mean ± SD of 6–7 animals) at 44–45 min post-injection of [^18^F]FDG. *P < 0.05 compared with conscious rats as determined using ANOVA with Dunnett’s multiple comparison test

**Fig. 3:**
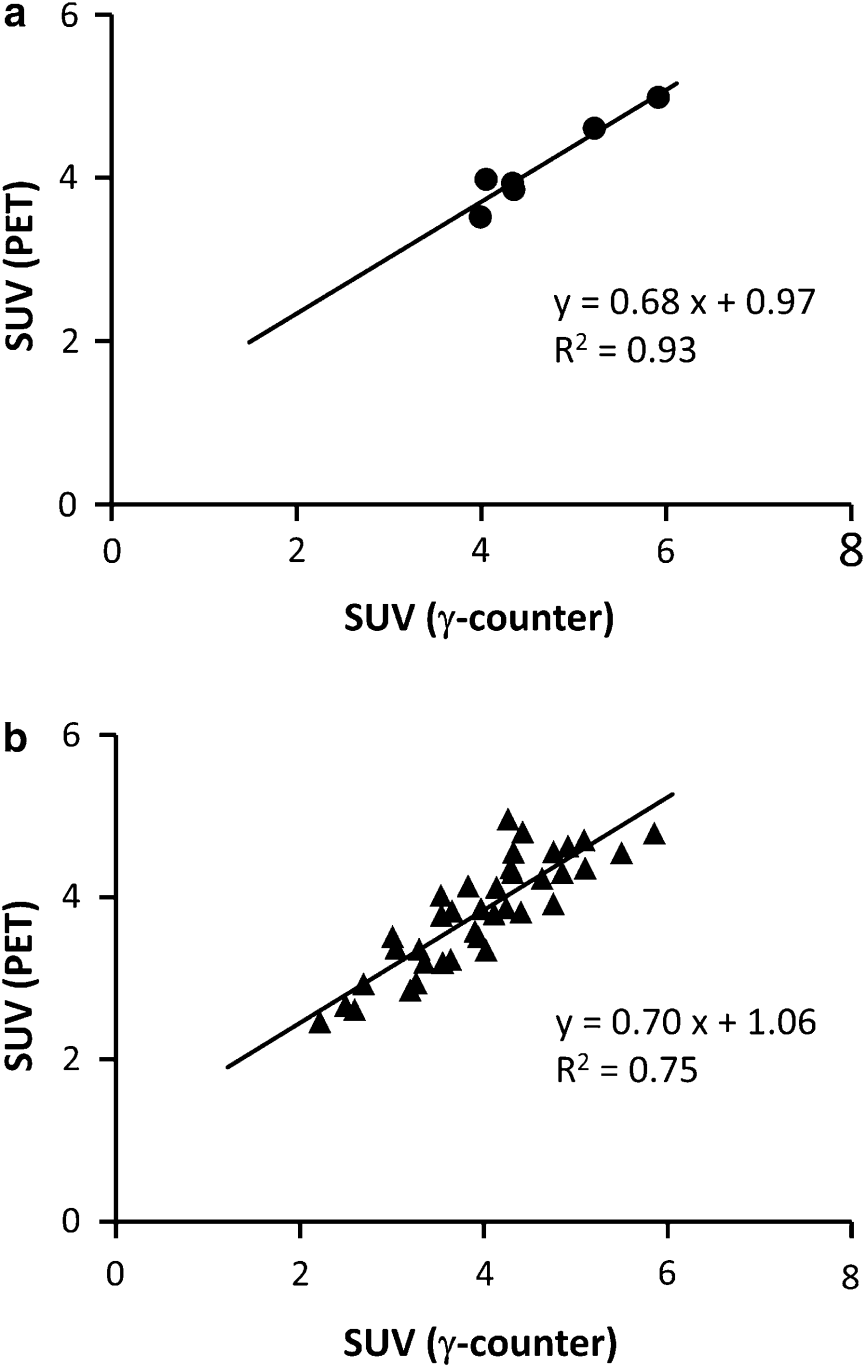
Quantitativity of conscious rat [^18^F]FDG-PET. **a** Correlation between SUV, determined by PET (44–45 min post-injection) and *γ* counter (~ 55 min post-injection) in conscious rats (n = 6), **b** Correlation between SUV, determined by PET (44–45 min post-injection) and *γ* counter (~ 55 min post-injection) in anesthetized rats (*n* = 38). Correlation coefficients (*R*) were calculated by Pearson’s product moment correlation coefficient

After acclimation to the restrainer, almost all (six in seven rats) conscious rats remained stable in the restrainer for at least 75 min. During PET/CT acquisition, the rats also remained stable without noticeable head movement, resulting in sufficient spatial resolution (Fig. 2). The quantitated brain uptake value of the last frame of [^18^F]FDG-PET (44–45 min post-injection) was well correlated with the [^18^F]FDG uptake determined using the γ counter after resection (~ 55 min post-injection) (Fig. 3a , *y* = 0.68 *x* + 0.97, *R*^2^ = 0.93, *P* < 0.01). The regression coefficient based on the correlation between conscious rat brain SUV determined by PET and by γ counter was not significantly different from that of anesthetized rats (Fig. 3, *t* = 0.023, *P* = 0.98).

The blood glucose levels and plasma corticosterone levels are summarized in Table 1. The arterial blood gas profiles are summarized in Additional file 1: Table S1. MMB- or KX-anesthetized rats showed significantly higher blood glucose levels than conscious rats (*P* < 0.01). The plasma corticosterone levels of conscious rats just before [^18^F]FDG injection were 10.4 ± 5.0 ng/mL (*n* = 4), and were not significantly different from those in MMB- and IFL-anesthetized rats (*P* > 0.05). The plasma corticosterone in conscious and MMB- and IFL-anesthetized rats remained low levels (< 20 ng/mL) during the 45-min [^18^F]FDG-PET acquisition and [^125^I]IMP assay, while slight increase in plasma corticosterone levels in conscious rats were observed during the 45-min [^18^F]FDG-PET acquisition (*P* < 0.05).

**Table 1:** Blood glucose levels and plasma corticosterone levels of conscious and anesthetized rats

	Blood glucose levels (mmol/L)	Plasma corticosterone levels (ng/mL)		
	Before [^18^F]FDG injection	Before [^18^F]FDG injection	After [^18^F]FDG-PET	After [^125^I]IMP assay
Conscious	7.51 ± 8.1	10.4 ± 5.0	13.6 ± 3.4^†^	15.9 ± 3.2^††^
MMB	10.0 ± 2.5**	9.42 ± 5.98	2.87 ± 3.59**	2.90 ± 4.79**
KX	10.6 ± 0.4**	n.d	n.d	n.d
Chloral	7.99 ± 1.35	n.d	n.d	n.d
PTB	6.30 ± 0.56	n.d	n.d	n.d
PF	6.42 ± 0.77	n.d	n.d	n.d
IFL	7.73 ± 0.81	8.94 ± 3.17	5.44 ± 2.66*	6.64 ± 3.96*

Data are expressed as mean ± SD (*n* = 6–7 for blood glucose levels, and *n* = 4 for plasma corticosterone levels). *P < 0.05 and **P < 0.01 compared with conscious rats as determined using ANOVA with Dunnett’s multiple comparison test. ^†^*P* < 0.05 and ^††^*P* < 0.01 compared with before [^18^F]FDG injection as determined using repeated ANOVA with Tukey’s multiple comparison test. *n.d.* not determined

### [^18^F]FDG kinetics in conscious and anesthetized rats

Typical [^18^F]FDG-PET images and the brain uptake values of conscious and anesthetized rats are summarized in Fig. 2. The whole-brain SUV of the IFL-anesthetized rats was significantly lower than that of the conscious rats (*P* < 0.05), and PET images showed marked reduction of [^18^F]FDG uptake in cerebrum and interbrain regions. There was a trend towards a small ubiquitous reduction in [^18^F]FDG in MMB- and Chloral-anesthetized rat brains (*P* > 0.05). KX-anesthetized rats showed low [^18^F]FDG uptake in cerebellum. The [^18^F]FDG distribution patterns in PTB- and PF-anesthetized rat brains were similar to those in conscious rat brains.

Additional file 1: Fig. S1 shows typical plasma and whole brain time activity curves (TACs) of [^18^F]FDG in conscious and anesthetized rats. MMB- and KX-anesthetized rats tended to show slow [^18^F]FDG clearance from the blood. The plasma SUV at 45 min post-injection in MMB- and KX-anesthetized rats were 1.39 ± 0.20 and 1.34 ± 0.34, respectively, and were significantly higher than those in conscious rats (0.84 ± 0.10, *P* < 0.01). Table 2 summarizes the [^18^F]FDG rate constants (*K*_1_, *k*_2_, *k*_3_, and *k*_4_) and the CMR_glu_ determined by TAC analysis. Although the *K*_1_, *k*_2_, and *k*_4_ values of the anesthetized rats were not significantly different from those of conscious rats (*P* > 0.05), there was a weak negative correlation between blood glucose levels and [^18^F]FDG *K*_1_ values (Additional file 1: Fig. S2). [^18^F]FDG phosphorylation reaction constants (*k*_3_) in all anesthetized rats were significantly lower than in conscious rats. The CMR_glu_ in conscious rats was higher than in the anesthetized rats (*P* < 0.05).

**Table 2:** [^18^F]FDG kinetics and cerebral metabolic rates of glucose (CMR_glu_) of conscious and anesthetized rats

	*K*_1_ (g/mL/min)	*k*_2_ (/min)	*k*_3_ (/min)	*k*_4_ (/min)	CMR_glu_ (mg/100 g/min)
Conscious	0.077 ± 0.015	0.119 ± 0.075	0.203 ± 0.156	0.012 ± 0.010	9.37 ± 1.85
MMB	0.086 ± 0.020	0.181 ± 0.076	0.089 ± 0.043**	0.001 ± 0.001	7.56 ± 0.93*
KX	0.065 ± 0.011	0.107 ± 0.030	0.058 ± 0.009**	0.001 ± 0.002	6.93 ± 0.61*
Chloral	0.105 ± 0.015	0.165 ± 0.052	0.072 ± 0.012*	0.001 ± 0.002	7.41 ± 1.15*
PTB	0.099 ± 0.020	0.151 ± 0.058	0.091 ± 0.036*	0.001 ± 0.001	6.68 ± 1.28**
PF	0.112 ± 0.024	0.219 ± 0.142	0.097 ± 0.060**	0.001 ± 0.001	6.22 ± 1.68**
IFL	0.102 ± 0.006	0.187 ± 0.044	0.062 ± 0.032**	0.001 ± 0.003	5.46 ± 1.69**

Data are expressed as mean ± SD (n = 6–7). *P < 0.05 and **P < 0.01 compared with conscious rats as determined using ANOVA with Dunnett’s multiple comparison test

**Fig. 4:**
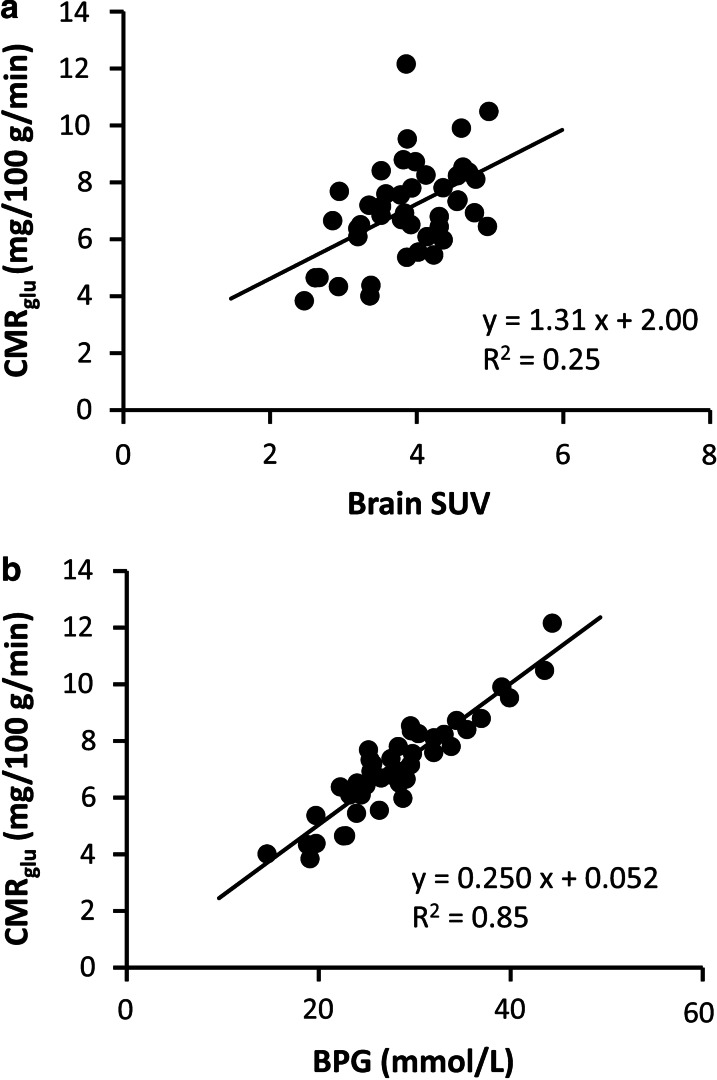
Correlation between [^18^F]FDG uptake and cerebral metabolic rates of glucose (CMR_glu_). **a** Correlation between SUV, determined by PET (44–45 min post-injection) and CMR_glu_ in conscious and anesthetized rats (*n* = 44). **b** Correlation between BPG and CMR_glu_ in conscious and anesthetized rats (*n* = 44). BPG was calculated by multiplying brain/plasma ratio by blood glucose levels. Correlation coefficients (*R*) were calculated by Pearson’s product moment correlation coefficient (*n* = 44)

The correlation between brain SUV at 45 min post-injection of [^18^F]FDG and CMR_glu_ was weak (Fig. 4a, *y* = 1.31 *x* + 2.00, *R*^2^ = 0.25, *P* < 0.01). BPG levels strongly correlated with CMR_glu_ (Fig. 4b, *y* = 0.0139 *x* + 0.0516, *R*^2^ = 0.85, *P* < 0.01).

**Fig. 5:**
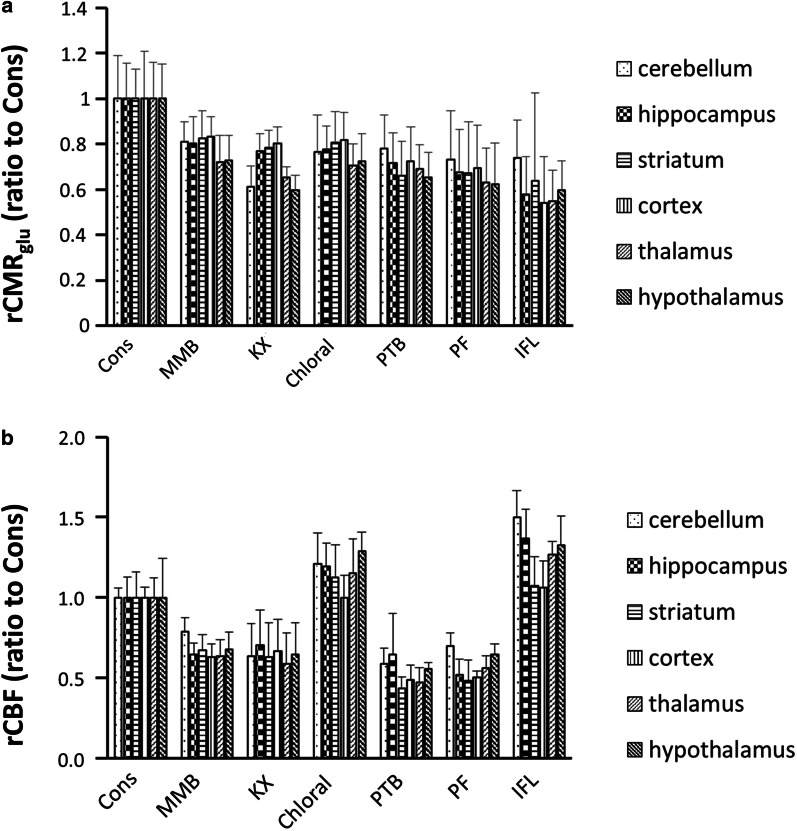
The effects of anesthetic agents on cerebral blood flow and metabolism. **a** The change in regional cerebral metabolic rates of glucose (rCMR_glu_) by anesthetic agents, calculated from [^18^F]FDG-dynamic PET. **b** The change in regional cerebral blood flow (rCBF) by anesthetic agents, determined using [^125^I]IMP. The values are expressed as ratio to values of conscious rats (mean ± SD, *n* = 6–7)

Regional CMR_glu_ (rCMR_glu_) is summarized in Fig. 5a. There was a ubiquitous reduction in rCMR_glu_ of MMB-, Chloral-, PTB-, and PF-anesthetized rats. KX-anesthetized rats showed highly reduced rCMR_glu_ in the cerebellum (*P* < 0.01) and interbrain (thalamus and hypothalamus, *P* < 0.01) compared with conscious rats. The reduction in cerebellum rCMR_glu_ in IFL-anesthetized rats was comparatively smaller than in other regions (*P* < 0.05 compared with cortex and thalamus).

### CBF determined using [^125^I]IMP

Table 3 shows the CBF determined using [^125^I]IMP. Conscious rats and Chloral-anesthetized rats showed similar CBF values. MMB-, KX-, PTB-, and PF-anesthetized rats showed lower CBF rates compared with conscious rats (*P* < 0.01). The CBF was higher in IF-anesthetized rats than in conscious rats (*P* < 0.01).

**Table 3:** Cerebral blood flow (CBF) in conscious and anesthetized rats, determined using [^125^I]IMP

	CBF (mL/100 g/min)
Conscious	94.7 ± 6.0
MMB	63.8 ± 8.1**
KX	62.5 ± 19.1**
Chloral	104.6 ± 12.9
PTB	49.3 ± 4.9**
PF	52.9 ± 4.4**
IFL	115.6 ± 8.4**

Data are expressed as mean ± SD (*n* = 6–7). **P < 0.01 compared with conscious rats as determined using ANOVA with Dunnett’s multiple comparison test

Regional CBF (rCBF) is summarized in Fig. 5b. MMB-, KX-, PTB-, and PF-anesthetized rats showed ubiquitously decreased rCBF in all brain regions. Relatively high rCBF was observed in the cerebellum, hippocampus, thalamus, and hypothalamus of Chloral- and IFL-anesthetized rats.

## Discussion

In the present study, the efficacy of the PET acquisition procedure using soft immobilization [11] for conscious rats (Fig. 1) was validated. This immobilization technique enabled 45-min dynamic PET acquisition of conscious rats without invasive surgery or the use of special techniques. The majority of rats were successfully habituated to the restrainer with simple immobilization procedures before the experiments. Only a few rats could not keep stable for 75 min in the restrainer. The plasma corticosterone levels, a stress marker for rodents, in conscious rats at just before [^18^F]FDG injection were not significantly different from those in MMB- and IFL-anesthetized rats, and were within the normal range [19]. Although the plasma corticosterone levels in conscious rats were slightly increased during the 45-min [^18^F]FDG-PET acquisition, the degree of increase was lower compared with traditionally reported restrainers [20–22], suggesting that restraint stress was minimal. Unelevated plasma corticosterone levels in MMB- and IFL-anesthetized rats between before [^18^F]FDG injection and after [^18^F]FDG-PET or [^125^I]IMP assay indicated that physical stress induced in our protocol was negligible. The brain PET images of conscious rats showed sufficient spatial resolution (Fig. 2). The brain SUV calculated from PET images (44−45 min post-injection) of conscious rats correlated well with that determined by γ counter after resection (~55 min post-injection), although the γ counter SUVs showed 1.1-times higher values than the PET images due to the difference in timing between PET and resection and/or attenuation (Fig. 3a). The regression coefficients of the regression lines of the conscious rats were not significantly different from that of the anesthetized rats (Fig. 3b), indicating that the effects of motion artifacts, and the attenuation and scattering by the restrainer were negligible. These results demonstrate that this conscious rat PET acquisition procedure using a soft restrainer could be widely applicable to neuronal PET imaging using various PET probes.

The CMR_glu_ in KX-, Chloral-, PTB-, PF-, and IFL-anesthetized rats was lower than that in conscious rats (Table 2). While the CMR_glu_ in the cerebellum of KX-anesthetized rats was markedly reduced, there was only moderate reduction of CMR_glu_ in the frontal cortex. Chloral, PTB, and PF ubiquitously reduced the CMR_glu_ in every region in the brain. Although IFL dramatically reduced the CMR_glu_ in the cerebrum and the interbrain regions, the reduction in CMR_glu_ in the cerebellum was slight (Fig. 5a). These findings are in good agreement with the previous reports [1, 5, 9, 23]. MMB is a mixed anesthetized agents that consists of an α_2_ adrenergic receptor agonist, a benzodiazepine-type GABA_A_ receptor modulator, and a κ-opiate receptor agonist [12]. MMB, as well as the other anesthetic agents, reduced rat CMR_glu_ (Table 2), and the reduction was distributed ubiquitously (Fig. 5a). Although the mechanism of the region-specific reduction in CMR_glu_ induced by anesthetic agents has not been fully elucidated, different reductions in CMR_glu_ between different brain regions could provide valuable information for designing neurobiological- and neurofunctional-experiments. [^18^F]FDG *k*_*3*_ in MMB-anesthetized rats as well as for rats with the other anesthetic agents-anesthetized rats was lower than that in conscious rats. The reduction in [^18^F]FDG phosphorylation is considered to be mainly caused by suppression of neuronal activity, although hexokinase activity might also be altered via the direct action of anesthetic agents [24, 25]. On the other hand, [^18^F]FDG *K*_*1*_ in all-anesthetized rats was similar to that in conscious rats, although speculatively [^18^F]FDG *K*_1_ could be reduced by saturation of glucose transporters with increased blood glucose in KX- and MMB-anesthetized rats. However, [^18^F]FDG *K*_1_ values were weakly and negatively correlated with blood glucose levels (Additional file 1: Fig. S2), indicating that the present sample size may not be large enough to detect the reduction in [^18^F]FDG *K*_1_ in KX- and MMB-anesthetized rats. Further studies are required to identify the detailed effects of anesthetic agents on [^18^F]FDG kinetics.

The CBF in MMB- as well as KX-, PTB-, and PF-anesthetized rats was also lower than that in conscious rats (Table 3), which could be coupled with CMR_glu_ reduction. In contrast, the CBF in Chloral-anesthetized rats was not significantly different from that in conscious rats, and IFL increased the CBF compared with conscious rats, resulting in uncoupling of CMR_glu_ and CBF (Tables 2, 3). These findings are consistent with those of previous reports [23, 26]. Uncoupling of CMR_glu_ and CBF in IFL-anesthetized rats might be intricately regulated by the CMR_glu_ decrease caused by an increase in cerebral lactate [27] and CBF increase caused by the vasodilation effect of IFL [28]. MMB could be effective for anesthetized animal neuroimaging because the effects of MMB on CMR_glu_ and CBF were comparatively small compared with those of the other anesthetized agents. However, MMB easily decreased the body temperature, probably due to the effects of medetomidine on central thermoregulation [29]. Because the CMR_glu_ and CBF were significantly lower in hypothermic rats than in normothermic rats (data not shown), the body temperature of MMB-anesthetized animals should be carefully controlled.

Anesthetic agents affect not only brain functions but also the whole-body physiological condition. For example, MMB- and KX-anesthetized rats showed higher blood glucose levels than conscious rats (Table 1) even after overnight fasting, which was reported to reduce the effects of anesthesia on blood glucose levels [30, 31]. This effect was possibly caused by the inhibition of insulin release on pancreatic islet cells by medetomidine and xylazine (α_2_ adrenergic receptor agonists) [32]. PET probe uptake in the brain, indicated by SUV, could be influenced not only by the brain functions but also by the whole-body physiologically condition. In the present study, brain SUV at 45 min post-injection could not accurately assess cerebral glucose metabolism (Fig 4a), which could be caused by delayed [^18^F]FDG clearance from blood circulation. These findings show that kinetic analysis with dynamic PET scan is generally necessary for the quantification of brain function in both anesthetized and conscious animals. Therefore, the soft immobilization technique presented here, which enables dynamic PET acquisition with/without arterial blood sampling, is a promising tool for noninvasive brain functional imaging studies using conscious rodents. On the other hand, BPG, which can be measured by static PET acquisition, was found to be well correlated with CMR_glu_ (Fig. 4b), suggesting its potential for utilization as an index of the brain glucose metabolic rate. Further studies are necessary to establish the applicability of BPG for the assessment of cerebral glucose metabolism.

## Conclusions

We have demonstrated the efficacy of soft immobilization for neuro-imaging of conscious rats. The facile and noninvasive technique enables dynamic PET acquisition without loss of spatial resolution and quantification, indicating this technique’s potential for neurofunctional imaging in conscious rodents.

## Supplementary Information

**Additional file 1: Supplementary Table S1**. Arterial blood gas profiles in conscious and anesthetized rats.** Supplementary Figure S1**. Typical time activity curves of [^18^F]FDG in the plasma and brain of conscious rats, and of rats anesthetized with different anesthetic agents: medetomidine/midazolam/butorphanol (MMB), ketamine/xylazine (KX), chloral hydrate (Chloral), pentobarbital (PTB), propofol (PF), and isoflurane (IFL). The asterisk and square indicate measured [^18^F]FDG SUV in the plasma and brain, respectively. Dotted and solid lines show calculated [^18^F]FDG SUV in plasma and brain by 2-tissue-3-compartment model analysis, respectively.** Supplementary Figure S2**. Correlation between blood glucose levels and [^18^F]FDG K1. Correlation coefficients (R = − 0.36728) were calculated by Pearson’s product moment correlation coefficient (n = 44).

## Data Availability

The datasets used and/or analysed during the current study are available from the corresponding author on reasonable request.

## Abbreviations

BPG: Index values, attained by multiplying the brain/plasma ratio by blood glucose levels
CBF: Cerebral blood flow
Chloral: Chloral hydrate
CMR_glu_: Cerebral metabolic rates of glucose
CONS: Conscious
[^18^F]FDG: 2-Deoxy-2-[^18^F] fluoro glucose
IFL: Isoflurane
KX: Ketamine + xylazine
MMB: Mixed anesthetic agent, which contain a mixture of medetomidine, midazolam, and butorphanol
MRI: Magnetic resonance imaging
n.d.: Not determined
PET: Positron emission tomography
PF: Propofol
PTB: Pentobarbital
rCMR_glu_: Regional cerebral metabolic rates of glucose
rCBF: Regional cerebral blood flow
ROI: Region of interest
SPECT: Single photon emission computed tomography
SUV: Standardized uptake value
TAC: Time activity curve
