# Supplementary material for: Conscious rat PET imaging with soft immobilization for quantitation of brain functions: comprehensive assessment of anesthesia effects on cerebral blood flow and metabolism
Source: EJNMMI Res. 2021 May 8;11:46. doi: 10.1186/s13550-021-00787-6 (PMC8106566; doi:10.1186/s13550-021-00787-6)

**Supplementary table S1**. Arterial blood gas profiles in conscious and anesthetized rats.

|  | **CONS** | **MMB** | **KX** | **Chloral** | **PTB** | **PF** | **IFL** |
| --- | --- | --- | --- | --- | --- | --- | --- |
| **pH** | 7.371 ± 0.049 | 7.337 ± 0.012 | 7.344 ± 0.035 | 7.313 ± 0.013 | 7.327 ± 0.064 | 7.346 ± 0.017 | 7.319 ± 0.053 |
| **pCO_2_ (mmHg)** | 50.4 ± 5.5 | 53.4 ± 3.3 | 49.7 ± 3.9 | 55.0 ± 3.1 | 59.0 ± 9.5 | 49.5 ± 5.6 | 58.0 ± 10.6 |
| **pO_2_ (mmHg)** | 90.3 ± 2.9 | 68.2 ± 7.2 | 79.7 ± 12.3 | 71.9 ± 8.9 | 70.7 ± 13.3 | 76.5 ± 5.5 | 192.9 ± 40.7 |

Data are expressed as mean ± SD (n = 6–7).

**Supplementary figure S1.** Typical time activity curves of [^18^F]FDG in the plasma and brain of conscious rats, and of rats anesthetized with different anesthetic agents: medetomidine/midazolam/butorphanol (MMB), ketamine/xylazine (KX), chloral hydrate (Chloral), pentobarbital (PTB), propofol (PF), and isoflurane (IFL). The asterisk and square indicate measured [^18^F]FDG SUV in the plasma and brain, respectively. Dotted and solid lines show calculated [^18^F]FDG SUV in plasma and brain by 2-tissue-3-compartment model analysis, respectively.


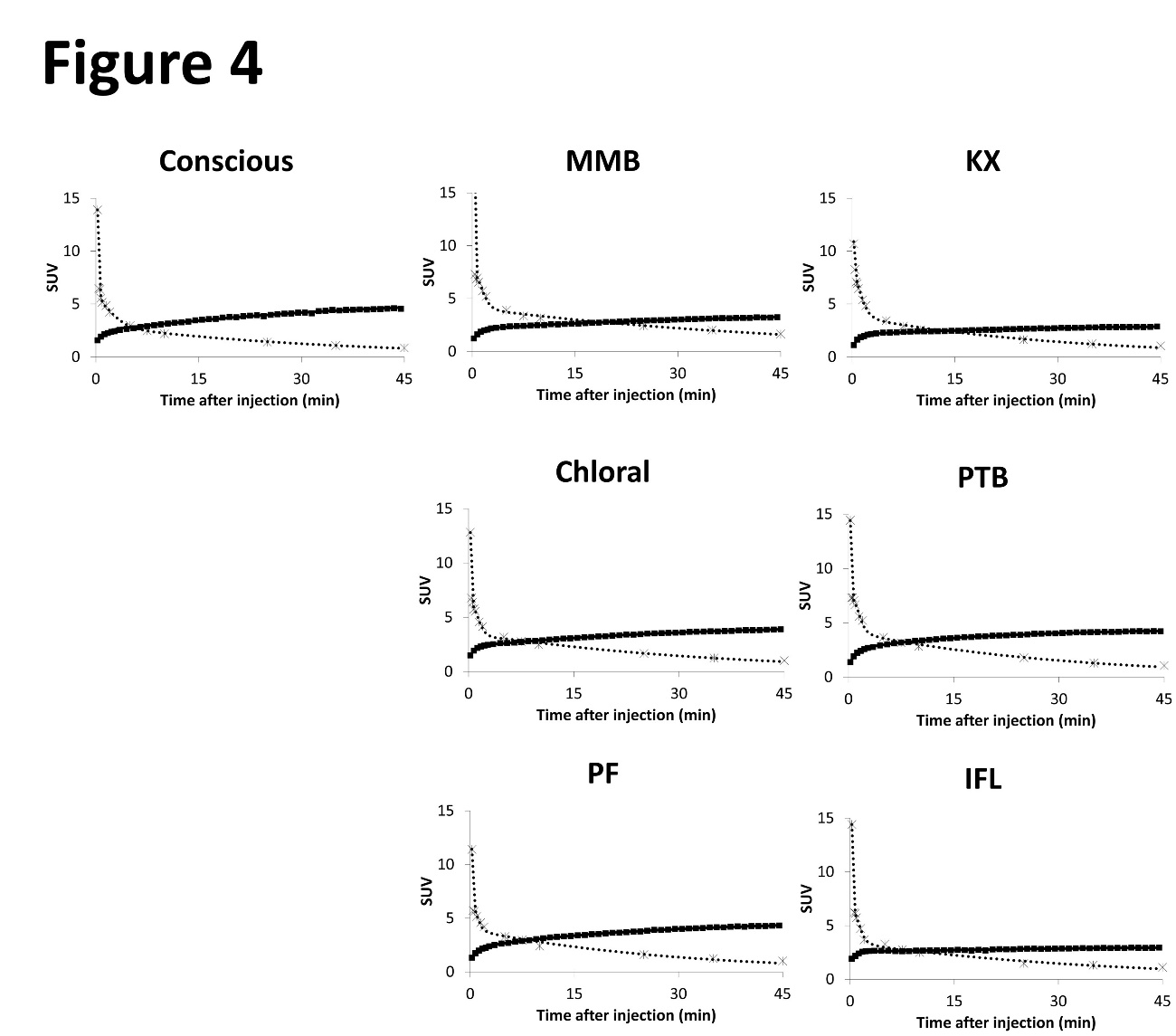


**Supplementary figure S2.** Correlation between blood glucose levels and [^18^F]FDG K_1_. Correlation coefficients (R = -0.36728) were calculated by Pearson’s product moment correlation coefficient (n = 44).


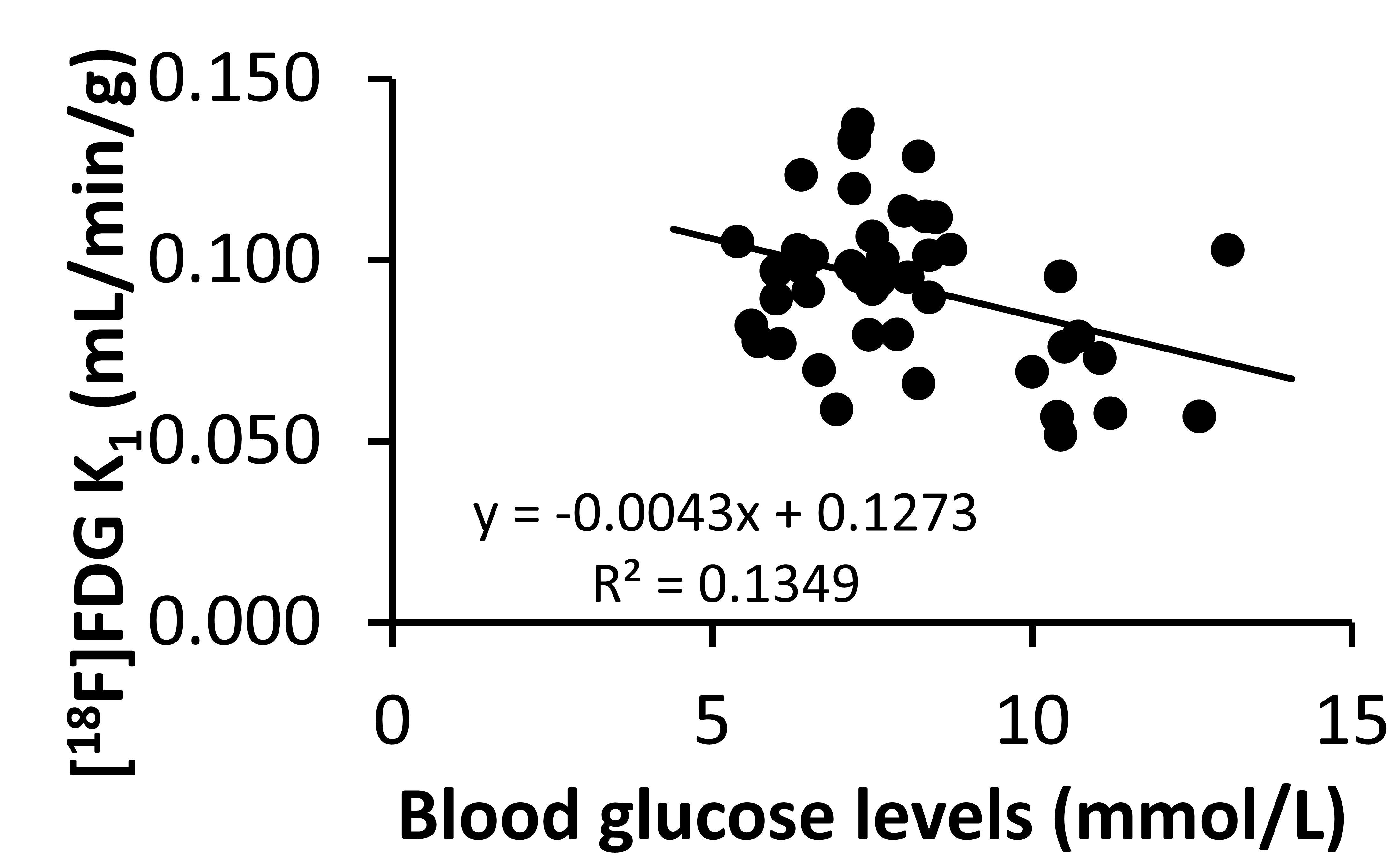

Supplement: Supplementary file 1 — Additional file 1: Supplementary Table S1. Arterial blood gas profiles in conscious and anesthetized rats. Supplementary Figure S1. Typical time activity curves of [18F]FDG in the plasma and brain of conscious rats, and of rats anesthetized with different anesthetic agents: medetomidine/midazolam/butorphanol (MMB), ketamine/xylazine (KX), chloral hydrate (Chloral), pentobarbital (PTB), propofol (PF), and isoflurane (IFL). The asterisk and square indicate measured [18F]FDG SUV in the plasma and brain, respectively. Dotted and solid lines show calculated [18F]FDG SUV in plasma and brain by 2-tissue-3-compartment model analysis, respectively. Supplementary Figure S2. Correlation between blood glucose levels and [18F]FDG K1. Correlation coefficients (R = − 0.36728) were calculated by Pearson’s product moment correlation coefficient (n = 44). [file 13550_2021_787_MOESM1_ESM.docx]
